# A rare case of extremely low birth weight infant with Beckwith-Wiedemann syndrome

**DOI:** 10.1016/j.ijscr.2024.109777

**Published:** 2024-05-19

**Authors:** Yuta Takeuchi, Seiichiro Inoue, Yuki Muta, Kohei Kawaguchi, Akio Odaka

**Affiliations:** Department of Pediatric Surgery, Saitama Medical Center, Saitama Medical University, 1981 Kamoda, Kawagoe, Saitama 350-8550, Japan

**Keywords:** Beckwith–Wiedemann syndrome, Extremely low birth weight infant, Omphalocele, Intestinal malrotation, Ladd surgery, Case report

## Abstract

**Introduction:**

Beckwith–Wiedemann syndrome (BWS) manifests distinctive features, such as macroglossia, overgrowth, and abdominal wall defects. In this report, we describe a case of BWS in an extremely low birth weight infant diagnosed at three months after birth because of the intensive care for low birth weight.

**Presentation of case:**

A female infant was delivered at 24 weeks and 6 days of gestation with a weight of 845 g. After birth, significant small intestinal intra-umbilical prolapse was observed, and abdominal wall closure using a sutureless method was performed on day zero. Careful neonatal management was performed; however, an episode of bloody stools led to a diagnosis of intestinal volvulus due to intestinal malrotation. At 119 days of age, the Ladd procedure was performed. Notably, during anaesthesia induction, features suggestive of BWS were observed, leading to its diagnosis.

**Discussion:**

Early diagnosis of BWS is vital because of its association with tumors. However, because she was an extremely low birth weight infant who required oral intubation and supine management for respiratory control, nevus flammeus and macroglossia were not observed. Therefore, BWS was not diagnosed for approximately three months after birth. It is important to recognize that omphalocele in extremely low birth weight infants is a risk factor for delayed diagnosis of BWS.

**Conclusion:**

Timely diagnosis of BWS is critical because of its association with tumors and varied clinical presentations. Early screening, especially for tumors, and awareness among surgical practitioners can aid in timely interventions and improved patient outcomes.

## Highlights

• Beckwith-Wiedemann Syndrome (BWS) is a rare genetic overgrowth condition present in neonates, and is associated with symptoms, including macroglossia, overgrowth, and abdominal wall defects.
• To avoid delay in diagnosis of BWS in extremely low birth weight, high index of suspicion is needed.
• This case underscores the importance of suspicion for BWS complications in patients with atypical presentations, such as extremely low birth weight and intestinal malrotation, as early diagnosis is key because of the association of BWS with development of tumours.

## 1 Introduction

Beckwith–Wiedemann syndrome (BWS) is the most common genetic overgrowth condition caused by genetic abnormalities involving chromosome 11p15, with a prevalence of approximately 1 in 10,000 live births [[1], [2], [3]]. The main signs include macroglossia, prenatal or postnatal overgrowth, and abdominal wall defects (omphalocele or umbilical hernia). Additionally, other features, including a linear groove in the auricle and small fossa at the posterior margin of the auricle, unilateral hypertrophy, neonatal hypoglycaemia, abdominal organ enlargement, renal malformations, and mid-glabellar capillary malformations (nevus flammeus), have been identified [4]. Therefore, several clinical diagnostic criteria are available for this condition [[4], [5], [6]]. Beckwith–Wiedemann syndrome is often diagnosed in the first month of life, particularly at birth [2]. However, BWS is strongly associated with the development of Wilms' tumor and hepatoblastoma [[7], [8], [9]], especially before 1 year of age; therefore, early diagnosis is important. In contrast, BWS is often associated with gigantism, and is relatively common in preterm infants; however, there are few reports of BWS in extremely low birth weight infants, which may pose a challenge in its diagnosis [10]. Furthermore, irrespective of BWS, these infants are perceived to be at an increased risk of hepatoblastoma compared to average weight infants [10], thereby underscoring the importance of early diagnosis.

In this report, we describe a case of BWS in an extremely low birth weight infant at the intensive care diagnosed three months after birth. This study was in line with the SCARE criteria [11].

## 2 Presentation of case

**Fig. 1:**
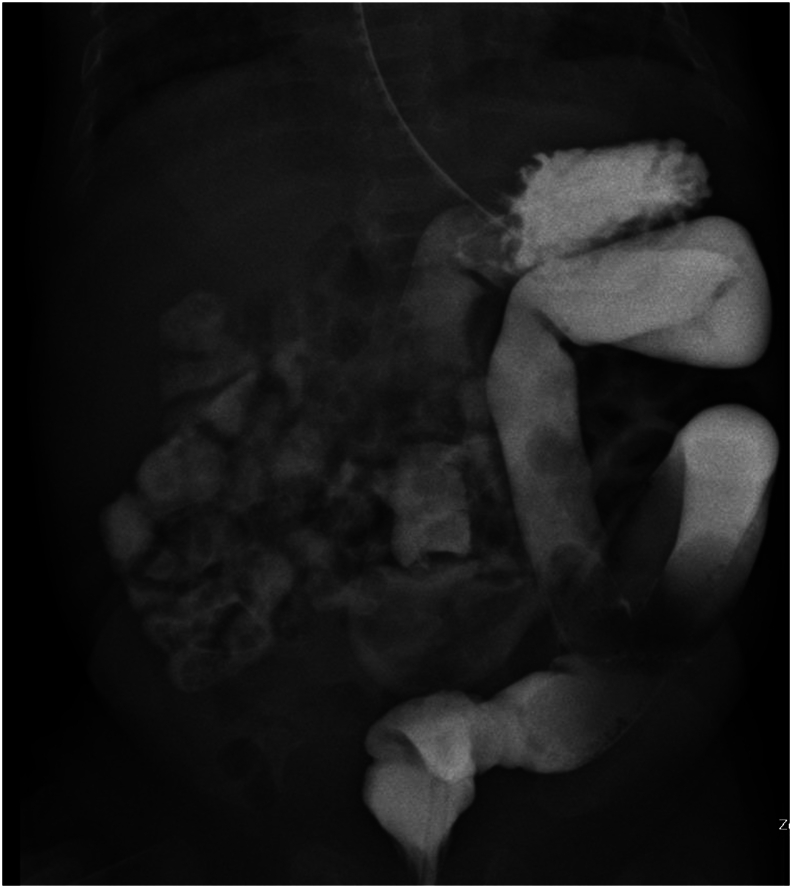
Upper and lower gastrointestinal tract contrast study The small intestine was on the patient's right side and the colon was on the left side, leading to the diagnosis of intestinal malrotation.

**Fig. 2:**
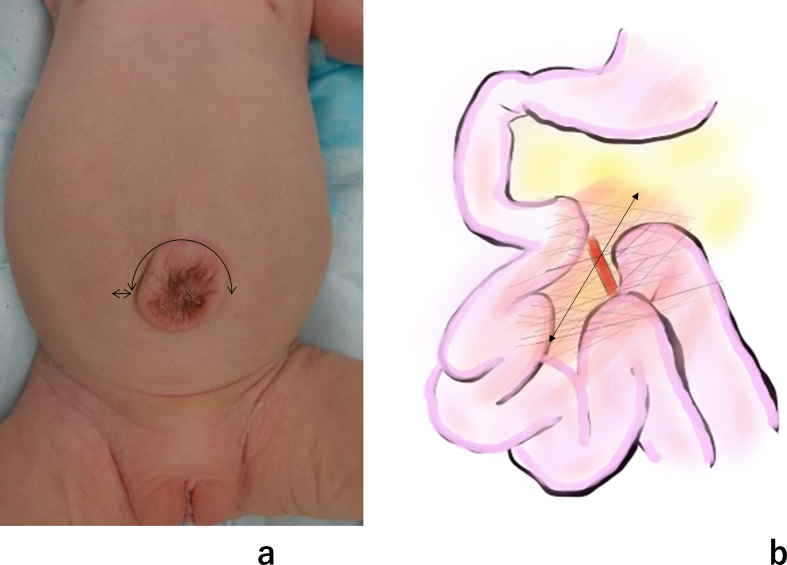
Surgical findings for abnormal bowel rotation (a) Skin incision was a supraumbilical arcuate incision slightly extended to the right(→). (b) Abnormal bowel rotation was confirmed, and the duodenum and small intestine were dissected because of an adhesion between the duodenum and small intestine in front of the superior mesenteric artery(→).

**Fig. 3:**
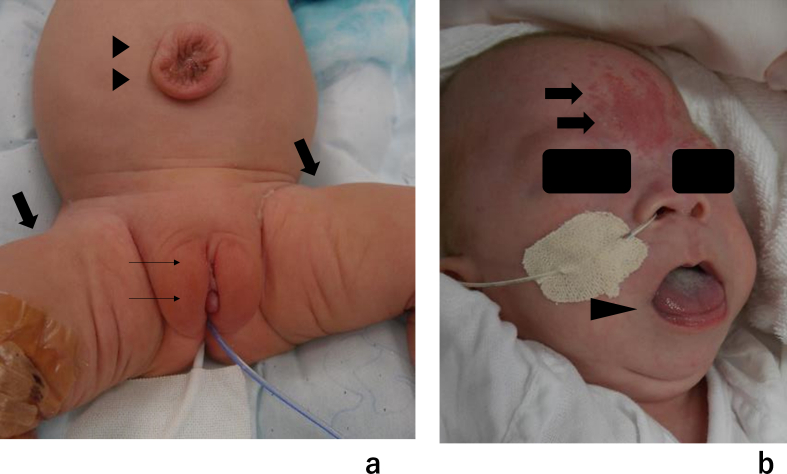
Photographs of the physical findings characteristic of BWS (a) Epithelialization was observed after an omphalocele procedure. Finally, the omphalocele was preoperative. Gigantism in the center of the lower leg and vulvar abnormalities (labia present, but penis-like protrusions) were noted. (b) Other findings included a macroglossia and flaming nevus.

A female infant was delivered via caesarean section at 24 weeks and 6 days of gestation due to placental insufficiency, with a birth weight of 845 g and Apgar scores of 2 at 1 min and 8 at 5 min. At 21 weeks and 4 days of gestation, foetal echocardiography revealed an abnormality in the abdominal wall. Subsequently, at 24 weeks and 6 days, an emergency caesarean section was necessary because of a decline in foetal heart rate. After birth, a large amount of small intestinal intra-umbilical cord prolapse was observed. Abdominal wall closure using the sutureless method was performed on day zero. Lifting of the umbilical cord allowed spontaneous return of the intestinal tract. The hernial sac was ligated close to the base of the umbilicus. At 15 days of age, excess hernia sacs were detached after ensuring epithelialisation. Due to an omphalocele, management was continued with a suspicion of congenital abnormality despite the baby's small size. On day 31, surgery was performed on open approach for patent ductus arteriosus. The patient was extubated at 46 days of age, and management continued in the supine position to stabilise his respiratory status. On day 91, the infant experienced apnoeic episodes and transient bloody stools. At 103 days of age, upper gastrointestinal tract contrast study and enteroscopy were performed, and intestinal malrotation was confirmed (Fig. 1). Consequently, the Ladd procedure was performed via an umbilical approach on day 119. The patient's abdomen was approached through a supraumbilical arcuate incision (Fig. 2). The duodenum and small intestine were detached due to adhesions, and appendicectomy was performed (Fig. 2). During anaesthesia induction, we observed an enlarged tongue and labia, which raised a suspicion of BWS (Fig. 3). The patient's postoperative course was uneventful and she was discharged 86 days after waiting for growth. Pre-discharge ultrasound examination revealed enlarged adrenal glands. She underwent regular outpatient ultrasonographic evaluations with no discernible enlargement, and her tumor markers exhibited no signs of deterioration.

## 3 Discussion

Beckwith–Wiedemann syndrome frequently presents during neonatal stages, exhibiting a diverse clinical spectrum characterised by varying degrees of severity. Macroglossia, a common manifestation of BWS, is observed in approximately 90 % of patients with BWS, and approximately 40 % of these children undergo lingual reduction surgery [5,12,13]. Therefore, patients with respiratory distress may require surgical intervention. In our case, although macroglossia was observed, respiratory symptoms were not severe at the time and did not necessitate surgery. Abdominal wall anomalies are also prevalent in most children afflicted with BWS, with overgrowth reported in approximately 40–60 % of cases [5]. In contrast, BWS has been infrequently reported in extremely low birth weight infants [10]. Other symptoms of neonatal hypoglycaemia in BWS result from excess insulin, which occurs in approximately 30–60 % of patients [14]. Also, the complication of hypoglycaemia in an umbilical cord omphalocele is important because it is life threatening immediately after birth [14]. The clinical diagnosis of BWS is based on these clinical symptoms and features. In our case, the patient was heavy for weight, but was an extremely low birth weight infant, and the giant body could not be identified. Additionally, she required management in the supine position with sedation for respiratory control; therefore, the nevus flammeus and macroglossia were not prominent. Differentiating BWS in extremely low birth weight infants is difficult because of its rarity. Therefore, the diagnosis of BWS was not made for approximately three months after birth until the time of surgery. Various factors have been reported to be associated with BWS; for example, assisted reproductive technologies have been demonstrated to potentiate the risk of developing BWS [12,15]. BWS is highly associated with tumors, and approximately 10 % of BWS cases are known to be complicated by some type of tumors, making early diagnosis desirable [7,8]. Specifically, Wilms tumor and hepatoblastomas affect approximately 2.5 % and 1.7 % of patients with BWS, respectively [7]. Furthermore, tracking alpha-fetoprotein (AFP) levels in patients with BWS is important because AFP levels associated with hepatoblastoma are essentially high [9,10]. Serial measurement of AFP levels early after birth is a valuable method for the early detection of hepatoblastoma [10]. Because of the time required for diagnosis in this case, AFP levels within the first 100 days of life were not measured. Therefore, continuous AFP levels from the early stage of the disease could not be confirmed. However, even now, at >1 year after birth, AFP values have not increased, and no tumor has been detected using ultrasound. The guideline for patients with BWS is based on a 3–6 months' abdominal ultrasound scanning and 2–3 months' αFP determinations up to 4–8 years of age [8]. Once a diagnosis is made, we can screen for tumors according to these guidelines [8].

However, although omphaloceles and intestinal malrotation are closely related [16], few reports have described an association between BWS and intestinal malrotation. Intestinal volvulus due to intestinal malrotation can present with symptoms such as haematochezia, which typically necessitates the Ladd procedure. In the present case, the patient was diagnosed with intestinal malrotation due to haematochezia, and surgical intervention was performed to stabilise the condition. Without confirmation of the patient's clinical characteristics at that time, the diagnosis of BWS may have remained elusive. Although our case was difficult to differentiate because of the extremely low birth weight of the infant, as a surgical practitioner, it is paramount to approach the patient with suspicion of potential BWS complications.

## 4 Conclusion

Beckwith–Wiedemann syndrome has a wide variety of symptoms; however, its diagnosis is important because of the tumor-related nature of the disease. As in our case, it would be helpful for the patient not to overlook casual symptoms at the time of surgery. Minor signs, including BWS, should not be overlooked when performing surgery for omphaloceles.

## Patient consent

Ethics approval and consent were waived because this case report is a review of literature with a retrospective case report on one patient. The patient gave consent to participate for publication. Written informed consent was obtained from the patient's parents/legal guardian for publication and any accompanying images. A copy of the written consent is available for review by the Editor-in-Chief of this journal on request. This report contains no personal information that could identify the patient.

## Ethical approval

The ethical committee approval was not required given the article type (case report). Ethical Approval: Not applicable.

## Funding

No funding or grant support.

## Author contribution

Conceptualization: Y.T, S.I, Y.M, K.K, A.O

Writing-original draft preparation: Y.T

Writing-review and editing: Y.T, S.I

## Guarantor

Yuta Takeuchi: Corresponding author.

## Research registration number

Ethics approval and consent were waived because this case report is a review of literature with a retrospective case report on one patient. The patient gave consent to participate for publication.

## Conflict of interest statement

The authors declare that they have no competing financial interests or personal relationships that may have influenced the work reported in this study.
